# The Synthetic Bacterial Lipopeptide Pam3CSK4 Modulates Respiratory Syncytial Virus Infection Independent of TLR Activation

**DOI:** 10.1371/journal.ppat.1001049

**Published:** 2010-08-19

**Authors:** D. Tien Nguyen, Lot de Witte, Martin Ludlow, Selma Yüksel, Karl-Heinz Wiesmüller, Teunis B. H. Geijtenbeek, Albert D. M. E. Osterhaus, Rik L. de Swart

**Affiliations:** 1 Department of Virology, Erasmus MC, University Medical Center, Rotterdam, The Netherlands; 2 EMC microcollections GmbH, Tübingen, Germany; 3 Academic Medical Center, University of Amsterdam, Amsterdam, The Netherlands; Vanderbilt University Medical Center, United States of America

## Abstract

Respiratory syncytial virus (RSV) is an important cause of acute respiratory disease in infants, immunocompromised subjects and the elderly. However, it is unclear why most primary RSV infections are associated with relatively mild symptoms, whereas some result in severe lower respiratory tract infections and bronchiolitis. Since RSV hospitalization has been associated with respiratory bacterial co-infections, we have tested if bacterial Toll-like receptor (TLR) agonists influence RSV-A2-GFP infection in human primary cells or cell lines. The synthetic bacterial lipopeptide Pam3-Cys-Ser-Lys4 (Pam3CSK4), the prototype ligand for the heterodimeric TLR1/TLR2 complex, enhanced RSV infection in primary epithelial, myeloid and lymphoid cells. Surprisingly, enhancement was optimal when lipopeptides and virus were added simultaneously, whereas addition of Pam3CSK4 immediately after infection had no effect. We have identified two structurally related lipopeptides without TLR-signaling capacity that also modulate RSV infection, whereas Pam3CSK4-reminiscent TLR1/2 agonists did not, and conclude that modulation of infection is independent of TLR activation. A similar TLR-independent enhancement of infection could also be demonstrated for wild-type RSV strains, and for HIV-1, measles virus and human metapneumovirus. We show that the effect of Pam3CSK4 is primarily mediated by enhanced binding of RSV to its target cells. The N-palmitoylated cysteine and the cationic lysines were identified as pivotal for enhanced virus binding. Surprisingly, we observed inhibition of RSV infection in immortalized epithelial cell lines, which was shown to be related to interactions between Pam3CSK4 and negatively charged glycosaminoglycans on these cells, which are known targets for binding of laboratory-adapted but not wild-type RSV. These data suggest a potential role for bacterial lipopeptides in enhanced binding of RSV and other viruses to their target cells, thus affecting viral entry or spread independent of TLR signaling. Moreover, our results also suggest a potential application for these synthetic lipopeptides as adjuvants for live-attenuated viral vaccines.

## Author Summary

Respiratory syncytial virus (RSV) infections are an important cause of hospitalization of infants during the winter season. However, RSV is often not the only detectable pathogen, but co-infections with respiratory bacteria are common. It has been hypothesized that this results from epithelial damage caused by the virus, facilitating colonization by pathogenic bacteria such as *Streptococcus pneumoniae*. However, an inverse order of events is not impossible: bacterial infections may activate respiratory epithelial cells through TLR signaling, resulting in increased susceptibility to virus infections. We tested this hypothesis by screening bacterial TLR agonists for their capacity to modulate RSV infection in different cell types, and identified the lipopeptide and prototype TLR1/2 agonist Pam3CSK4 as an enhancer of RSV infections. However, to our surprise this proved independent of TLR activation, but was mediated by enhancement of binding between virus and target cell. Two structurally related lipopeptides unable to stimulate TLR responses were identified that enhanced infections with RSV, but also with other enveloped viruses including HIV-1, human metapneumovirus, and measles virus. We speculate that bacterial infections may influence the pathogenesis of virus infections by facilitating binding to target cells.

## Introduction

Human respiratory syncytial virus (RSV) is a major cause of respiratory tract disease in infants, immunocompromised subjects and the elderly [1]. The virus is a member of the family *Paramyxoviridae*, which also includes human metapneumovirus (HMPV) and measles virus (MV). RSV owes its name to the formation of multinucleated syncytia within infected epithelial cells of the respiratory tract [2]–[4]. RSV shows a seasonal epidemiology associated with worldwide peaks in virus transmission during the winter or rainy season [5]. In most cases the virus causes a mild and self-limiting upper respiratory tract infection. However, in some cases (usually estimated as 1–2%) the virus spreads to the lower respiratory tract, and may cause severe bronchiolitis or pneumonia [1], [5]. A substantial proportion of these patients require hospitalization, and occasionally mechanical ventilation.

Risk factors for developing severe RSV disease include premature birth, immune deficiency, underlying chronic lung disease or congenital heart disease [1], [5]. However, in the majority of hospitalized cases no risk factor can be identified. The pathogenesis of these severe RSV cases remains poorly understood. Different explanations have been proposed, such as anatomical predispositions, mucus overproduction, skewed T-helper 2 immune responses or co-infections. Some studies have suggested that co-infections by RSV and the closely related HMPV may result in severe disease [6], [7], but co-infections with bacteria or other respiratory viruses have also been described, especially for *Streptococcus pneumoniae* (SP) [8]–[11]. Invasive pneumococcal disease has been shown to be more prevalent during the RSV season [12]. In addition, the frequency of hospitalization for severe RSV disease is reduced in children who have been vaccinated against SP [13].

In addition to SP, different bacteria have been detected in nasal swabs, nasopharyngeal aspirates or broncho-alveolar lavages of children with severe RSV infections, including *Staphylococcus aureus* (SA), *Haemophilus influenza* (HI) *and Moraxella catarrhalis* (MC). RSV-infected children are often co-diagnosed with otitis media caused by SP, HI or MC [14]. It is often assumed that viral infections precede superinfection with bacteria [15]–[17], by causing epithelial damage that allows bacterial colonization or by facilitating bacterial binding to epithelial cells [18]–[20]. However, an inverse order of events cannot be excluded: respiratory bacteria may facilitate virus infections by activating target cells or modulating virus-specific immune responses [21].

The mammalian immune system has developed pattern recognition molecules such as Toll-like receptors (TLRs) [22], which are not only expressed by professional antigen-presenting cells but also by epithelial cells of the respiratory tract [23]–[25]. TLR triggering by pathogens, including bacterial structures, leads to an innate and adaptive immune response to specifically combat the invading pathogen. However, by changing the phenotype of the cell, TLR signaling might also increase the susceptibility of cells to virus infection. For HIV-1 it has been described that bacterial TLR ligands enhance infection of and transmission to target cells [26]–[30]. Here, we have examined the effect of bacterial TLR ligands and structurally related molecules on RSV infection in different cell types.

## Results

### The lipopeptide and prototype TLR1/2 agonist Pam3CSK4 modulates RSV infection of epithelial and antigen presenting cells

**Figure 1:**
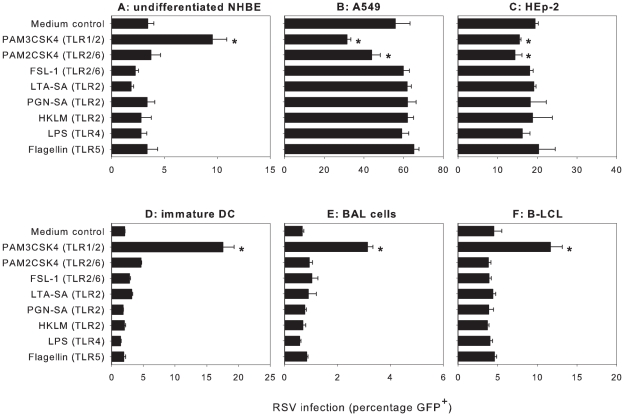
Pam3CSK4 modulates rgRSV infection in different cell types. (A–F) Primary cells or immortalized cell-lines were incubated with bacterial TLR ligands using conditions as described previously [28]. In undifferentiated primary normal human bronchial epithelial (NHBE) cells, antigen presenting cells (immature DC, BAL cells and EBV-transformed B-LCL), Pam3CSK4 mediated significant enhancement of rgRSV infection (*p<0.05 in 1-way ANOVA and posthoc Dunnett's correction for multiple comparisons with medium control). In contrast, treatment of A549 or HEp-2 cells with Pam3CSK4 and Pam2CSK4 resulted in reduced rgRSV infection levels (*p<0.05 using Dunnett's correction). Data are presented as percentages GFP-positive cells (means ± SD of triplicates). A representative experiment out of three is shown.

The TLR family consists of more than ten members, each interacting with specific pathogenic structures [31]. TLR1, 2, 4, 5 and 6 have been shown to interact with bacterial structures. A panel of prototype bacterial TLR ligands was tested for their ability to modulate RSV infection of different target cells. Both primary cells and immortalized cell lines were used, since TLR expression is influenced by the activation status of cells. The epithelial cells, the main target cells for RSV infection, were the initial focus of our investigation. Following a previously described protocol [28], cells were pre-incubated with the respective TLR ligands and subsequently infected with a recombinant RSV (strain A2) that encodes enhanced GFP (rgRSV) [32]. In primary undifferentiated normal human bronchial epithelial (NHBE) cells, pre-incubation with the synthetic bacterial lipopeptide Pam3-Cys-Ser-Lys4 (Pam3CSK4) enhanced rgRSV infection (p<0.05), whereas the other TLR ligands did not modulate infection (Figure 1A). Pam3CSK4 was also found to enhance rgRSV infection in well-differentiated NHBE cells grown on air-liquid interface (results not shown). The epithelial cell lines A549 and HEp-2 are frequently used to study RSV infection and the percentage of infected cells is higher compared to the primary NHBE cells (Figure 1A). Surprisingly, and in contrast to NHBE cells, Pam3CSK4 and to a lesser extent Pam2CSK4 did not enhance but rather decreased rgRSV infection in A549 and HEp-2 (p<0.05) (Figure 1B, C).

Antigen presenting cells (APC) in the respiratory tract, such as alveolar macrophages and dendritic cells, are exposed to RSV during infection. The level of infection of these cells *in vivo* and the contribution of these cells to RSV pathogenesis is currently unknown. Since APC are highly responsive to TLR agonists, we studied infection of primary antigen presenting cells, including monocyte-derived dendritic cells and BAL cells, in the presence and absence of TLR agonists. In addition, we also used an Epstein-Barr virus-transformed B-lymphoblastic cell line (B-LCL) representing RSV infection of lymphoid APC. APC can be infected with RSV but are relatively resistant compared to epithelial cells. Using a 5-fold higher dose in immature monocyte-derived dendritic cells (DCs) (Figure 1D) and a 10-fold higher dose in BAL cells and B-LCL (Figure 1E,F), infection with rgRSV resulted in 2%, 1%, and 5% GFP-expressing cells, respectively. Similar to NHBE cells, pre-incubation of APC with Pam3CSK4 strongly increased rgRSV infection levels in these cells (p<0.05) (Figure 1D-F). These results demonstrate that the TLR1/TLR2 ligand Pam3CSK4 modulates rgRSV infection of different target cells.

### Pam3CSK4-mediated enhancement of RSV infection in APC is a rapid and dose-dependent process

**Figure 2:**
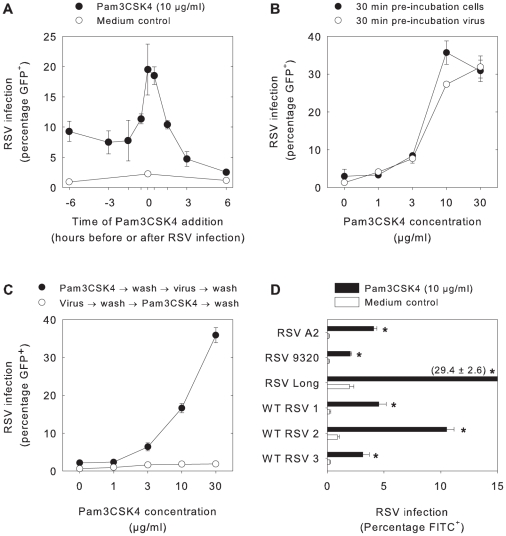
Pam3CSK4 mediates rapid and dose-dependent enhancement of RSV infection in B-LCL. (A) B-LCL were incubated at 37°C with Pam3CSK4 (10 µg/ml) at 6 h, 3 h, 1.5 h, 0.5 h or 0 h before or 0.5 h, 1.5 h, 3 h, and 6 h after rgRSV infection. Pam3CSK4 treatment resulted in significantly increased rgRSV infection percentages in B-LCL at all time points (*p<0.005 with Student's t-tests with Bonferroni correction for multiple comparisons). (B) B-LCL or virus were pre-incubated with different concentrations of Pam3CSK4 for 30 minutes at 37°C before infection with rgRSV. Pam3CSK4 treatment resulted in significantly increased rgRSV infection percentages (p<0.05 for Pam3CSK4 concentrations ≥3 µg/ml), but no biologically relevant differences were detected between pre-incubation of cells or virus. (C) Cells were treated with different concentrations of Pam3CSK4 either directly before or after rgRSV infection. In all conditions free lipopeptide or free virus was washed away before proceeding to the next step. Pam3CSK4 treatment resulted in significantly higher percentages of infected cells (p<0.01, significant under Bonferroni correction for Pam3CSK4 concentrations ≥3 µg/ml). However, addition of Pam3CSK4 after rgRSV infection and subsequent removal of free virus had no effect. (D) B-LCL were incubated for 30 minutes at 37°C with or without Pam3CSK4 (10 µg/ml) before infection with different RSV strains. Pam3CSK4 treatment resulted in increased RSV infection percentages (p<0.05) for all strains tested. In all panels data are presented as percentages RSV-infected cells (means ± SD of triplicates) 20–24 hours after infection as determined by flow cytometry, using GFP expression in panels A–C and staining with FITC-labeled RSV-specific antibodies in panel D. Representative experiments out of three are shown.

To further investigate the mechanism by which Pam3CSK4 enhances RSV infection, the optimal time for addition of the lipopeptide before or after infection was determined. For these studies B-LCL were used, as these are easier to work with than the primary epithelial cells or myeloid APC and thus facilitated further experiments. At different time points before or after infection with rgRSV B-LCL were incubated with Pam3CSK4. Interestingly, addition of the lipopeptides at the same time as the virus resulted in the highest percentages of infected cells (p<0.005, Figure 2A). This result suggests that Pam3CSK4 directly influences binding or entry of the virus. A similar enhancement of rgRSV infection could also be demonstrated using alternative readout parameters, including measurement of virus titers in supernatant by endpoint titration or assessment of numbers of infected cells by infectious centre assay (data not shown).

Subsequently, RSV or B-LCL were pre-incubated with different concentrations of Pam3CSK4 for 30 min at 37°C before infection and overnight culture. A convincing and dose-dependent enhancement of infectivity was observed in both conditions (p<0.05, Figure 2B). At 30 µg/ml Pam3CSK4 became toxic for the cells, resulting in a declining percentage of infected cells especially in the condition where cells had been exposed directly. To discriminate between pre- and post-entry mechanisms, B-LCL were incubated with different concentrations of Pam3CSK4 directly before or after rgRSV infection. In B-LCL incubated with Pam3CSK4 and washed before rgRSV infection, enhancement of infection was still detectable (p<0.01, Figure 2C), suggesting that cell-bound Pam3CSK4 directly or indirectly increased rgRSV infection. In contrast, Pam3CSK4 did not influence the percentage of GFP-expressing cells when the lipopeptide was added immediately after rgRSV infection and subsequent washing to remove unbound virus, suggesting that Pam3CSK4 does not affect post-binding or -entry steps of RSV infection, such as transcription, replication, release and second round infection. In addition, as Pam3CSK4 was washed away the toxic effect was not present anymore, resulting in enhancement for all tested concentrations. Moreover, the level of GFP expression in Pam3CSK4-stimulated cells was similar to GFP expression in untreated cells, suggesting that RSV transcription was not influenced (data not shown). In order to exclude that the observed effect was related to a specific property of the recombinant RSV strain used in these experiments, we also tested the effect of Pam3CSK4 on infection of B-LCL with different laboratory or wild-type strains of RSV, which resulted in a similar enhancement of infection (p<0.05, Figure 2D). Thus, we conclude that Pam3CSK4 enhances RSV binding or entry in B-LCL. These findings were corroborated in primary epithelial cells and DCs (data not shown).

### Pam3CSK4 enhances RSV binding to target cells independent of TLR signaling

**Figure 3:**
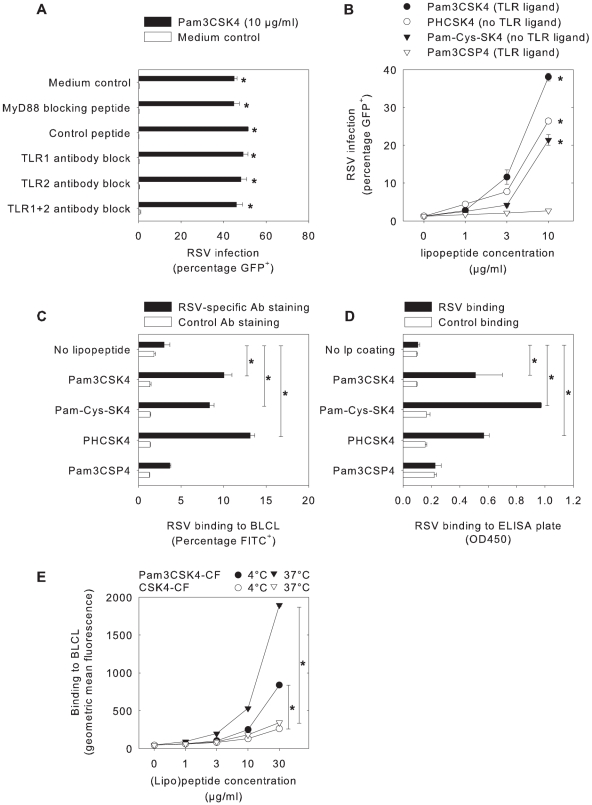
The effect of Pam3CSK4 on B-LCL is independent of TLR signaling, but mediated by enhanced virus binding. (A) B-LCL were pre-incubated with the myeloid differentiating factor 88 (MyD88) homodimerization inhibitory peptide, a control peptide, or polyclonal blocking antibodies to TLR1, TLR2 and/or TLR4, followed by Pam3CSK4 incubation (10 µg/ml, 30 minutes, 37°C) and subsequent infection with rgRSV. In all conditions Pam3CSK4 treatment resulted in enhancement of rgRSV infection (p<0.05). (B) B-LCL were incubated with different lipopeptides (for molecular structures see Figure S1) with or without TLR signaling capacities. In addition to TLR agonist Pam3CSK4 two non-TLR2 activating lipopeptides Pam-Cys-SK4 and PHCSK4 enhanced rgRSV infection, whereas the TLR agonist Pam3CSP4 did not (p<0.05 with Dunnett's correction for multiple comparisons for Pam3CSK4, Pam-Cys-SK4, and PHCSK4 concentrations 3 and 10 µg/ml). (C) RSV binding to B-LCL was determined by pre-incubating rgRSV with the different lipopeptides, followed by assessment of binding to cells. RSV binding was quantified by staining with FITC-labeled antibodies to RSV or to influenza-B as a control. Addition of the lipopeptides Pam3CSK4, Pam-Cys-SK4 and PHCSK4 but not Pam3CSP4 significantly increased binding of RSV to the cells (p<0.05 using Dunnett's correction for multiple testing). (D) Direct RSV binding to lipopeptides was examined in an ELISA format. Lipopeptides were coated on high-binding ELISA plates and incubated with RSV, or with MV strain Edmonston as a control. RSV binding was measured using an RSV-specific monoclonal antibody, followed by detection using a goat-anti-mouse peroxidase and TMB as a substrate. Coating with Pam3CSK4, Pam-Cys-SK4 and PHCSK4 but not with Pam3CSP4 resulted in increased RSV binding (p<0.05 compared with Dunnett's correction). Data are shown as extinctions at 450 nm (means ± SD of duplicates). (E) B-LCL were incubated with Pam3CSK4-CF or CSK4-CF at 4°C or 37°C to measure direct (lipo)peptide binding to the cells. At both temperatures Pam3CSK4-CF showed significantly stronger binding than CSK4-CF (p<0.01 with Bonferroni correction at concentrations 10 and 30 µ/ml). In panels A-C results are shown as percentages GFP- or FITC-positive cells (means ± SD of duplicates) and in Panel E data are shown as geometric mean fluorescence (means ± SD of triplicates). Representative examples of at least three experiments are shown.

Since the addition of Pam3CSK4 together with RSV had the largest influence on rgRSV infection, we hypothesized that TLR triggering and subsequent phenotypical changes might not be involved in this effect. Indeed, neither blocking of Pam3CSK4 binding to TLR by neutralizing antibodies nor blocking of TLR signaling by blocking peptides abrogated enhancement of rgRSV infection by Pam3CSK4 (p<0.05, Figure 3A). To further support these results, different lipopeptides were used that were structurally similar to Pam3CSK4 but do not contain TLR signaling capacities (see Figure S1 for molecular structures). Pam-Cys-SK4 and PHCSK4 do not induce TLR signaling [33], [34], but enhanced rgRSV infection in B-LCL (p<0.05, Figure 3B). Furthermore, Pam3CSP4, which is equally effective in inducing TLR responses as Pam3CSK4 [35], did not enhance rgRSV infection under these conditions (Figure 3B). Thus, Pam3CSK4 enhances RSV infection independently of TLR signaling.

To investigate whether Pam3CSK4, Pam-Cys-SK4 and PHCSK4 enhance attachment of rgRSV to target cells, an RSV binding assay was performed. In contrast to Pam3CSP4, pre-incubation of rgRSV with Pam3CSK4, Pam-Cys-SK4 or PHCSK4 increased binding to B-LCL (p<0.05, Figure 3C). Subsequently, the direct interaction of lipopeptides and viruses was investigated in an RSV binding ELISA. The different lipopeptides were coated onto ELISA plates and subsequently incubated with rgRSV. Pam3CSK4, Pam-Cys-SK4 and PHCSK4, but not Pam3CSP4, were found to bind rgRSV (p<0.05, Figure 3D). Finally, B-LCL were incubated with the fluorescent (lipo)peptides Pam3CSK4-CF or CSK4-CF at 4°C or 37°C, demonstrating effective membrane binding of Pam3CSK4-CF at concentrations that were also found to enhance rgRSV infection (p<0.01, Figure 3E). Together these results demonstrate that Pam3CSK4 interacts directly with rgRSV or its target cells, enhances binding of rgRSV to target cells and thereby increases infection efficiency, all independent of TLR signaling.

### Charge and structure of lipopeptides play an essential role for enhancement

**Figure 4:**
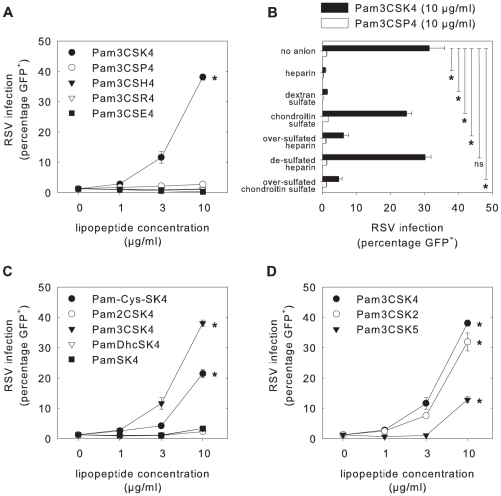
Importance of lipopeptide charge and structure. (A, C, D) B-LCL were incubated with different lipopeptides for 30 minutes at 37°C before infection with rgRSV. Only Pam3CSK4, Pam-Cys-SK4, Pam3CSK2 and Pam3CSK5 resulted in enhancement of rgRSV infection, whereas Pam3CSE4 resulted in inhibition of rgRSV infection compared to medium control (p<0.05 in 1-way ANOVA and posthoc Dunnett's correction). (B) Natural and chemically-derived polyanionic compounds were assessed to block the Pam3CSK4-mediated enhancement of RSV infection. The compounds were added to cells that had been pre-incubated with Pam3CSK4 or Pam3CSP4 as a control, followed by infection with RSV. The next day percentages GFP-expressing cells were determined by flow cytometry. The strongly polyanionic compounds heparin, dextran sulfate, over-sulfated heparin and over-sulfated chondroitin sulfate were able to abrogate the Pam3CSK4-mediated enhancement of rgRSV infection (p<0.05 using posthoc Dunnett's correction), whereas the control compounds chondroitin sulfate and de-sulfated heparin had only a minimal effect or no effect, respectively. Error bars represent the standard deviation of triplicates. A representative experiment out of three is shown.

Charge and structure can play important roles in molecular interactions. Several cationic molecules have been described to enhance viral infections [36]–[38]. To determine the role of the four lysines in Pam3CSK4, which are strongly cationic, these were replaced by either the neutral amino acid proline (Pam3CSP4), the weakly cationic histidine (Pam3CSH4), the strongly cationic arginine (Pam3CSR4), or the negatively charged glutamic acid (Pam3CSE4) (see Figure S1 for molecular structures). Of these lipopeptides, only Pam3CSK4 was able to enhance rgRSV infection (Figure 4A). Despite their positive charge, neither Pam3CSH4 nor Pam3CSR4 were capable of enhancing RSV infection. In contrast to the other four lipopeptides, Pam3CSE4 reproducibly inhibited RSV infection (p<0.05).

To explore the importance of electrostatic interactions between Pam3CSK4 and cells or virus, various polyanionic compounds were tested to shield the positive charge of Pam3CSK4, using previously described conditions [38]. Two naturally occurring polyanionic compounds, heparin and chondroitin sulfate, and four chemically desulfated or oversulfated anionic polymers, dextran sulfate, over-sulfated heparin, de-sulfated heparin and oversulfated chondroitin sulfate, were tested for their effect on the interaction between Pam3CSK4 and RSV. Desulfated or oversulfated anionic polymers were used to normalize for differences in relative anionic strengths between the naturally existing compounds. After incubation of B-LCL with Pam3CSK4, heparin, dextran sulfate, or chondroitin sulfate were added, resulting in a decreased levels of rgRSV infection after treatment with heparin and dextran sulfate (p<0.05), whereas chondroitin sulfate did not effectively block rgRSV infection (Figure 4B). Oversulfated heparin and oversulfated chondroitin sulfate inhibited Pam3CSK4-mediated enhancement of RSV infection (p<0.05), but desulfated heparin did not. In addition, RSV binding experiments using B-LCL incubated with Pam3CSK4 showed decreased virus binding after treatment with heparin, dextran sulfate, oversulfated heparin or over-sulfated chondroitin sulfate, but not after treatment with chondroitin sulfate or de-sulfated heparin (data not shown). These results suggest that highly sulfated polyanions neutralize the cationic property of the cell-bound Pam3CSK4, resulting in abrogation of the Pam3CSK4-mediated enhancement of RSV binding and infection, and confirm that cationic properties of Pam3CSK4 are crucial for their capacity to enhance RSV infections.

Subsequently, other properties of the lipopeptide structure were studied. Two lipopeptides with either one (Pam-Cys-SK4), two (Pam2CSK4) or three (Pam3CSK4) palmitoyl residues were tested for their capacity to enhance rgRSV infection in B-LCL (see Figure S1 for molecular structures). Pam-Cys-SK4 strongly enhanced rgRSV infection (p<0.05), whereas Pam2CSK4 had limited to no enhancing properties, demonstrating that the amino-linked palmitoyl group is crucial for the enhancing properties (Figure 4C). In addition, a Pam-Cys-SK4-reminiscent lipopeptide, containing the prokaryotic amino acid S-(2,3-dihydroxypropyl)cysteine (Dhc) characterized by two free hydroxyl groups (PamDhcSK4), and a lipopeptide completely lacking the cysteinyl partial structure (PamSK4) were tested, which both were unable to enhance infection. These results demonstrated that the amphiphilic feature and cationic lysins per se are insufficient for enhancement, and strongly suggest that the N-palmitoylated cysteine is necessary as membrane anchor. Evaluation of Pam3CSK4-reminiscent lipopeptides with two or five instead of four lysins (Pam3CSK2 and Pam3CSK5, respectively) showed that all could enhance rgRSV infection in B-LCL (p<0.05, see Figure S1 for molecular structures). However, the level of enhancement was not simply related to the number of cationic lysins in the lipopeptide, as Pam3CSK2 showed a stronger enhancement than Pam3CSK5 (Figure 4D).

### Pam3CSK4, Pam-Cys-SK4 and PHCSK4 also enhance infection with other enveloped viruses

**Figure 5:**
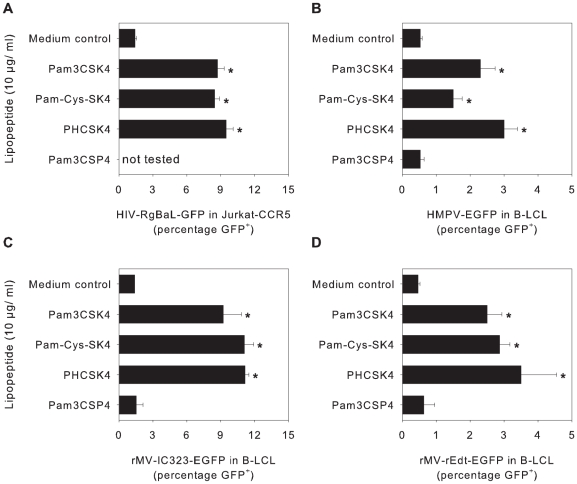
Enhancement of infection with other enveloped viruses. (A–D) Jurkat-CCR5 cells (A) or B-LCL (B–D) were incubated with different lipopeptides (10 µg/ml, Pam3CSK4, Pam-Cys-SK4, PHCSK4, or Pam3CSP4) before infection with HIV-RgBaL-GFP (A), HMPV-EGFP (B), rMV-Edmonston-EGFP (C) or rMV-IC323-EGFP (D), respectively. Two (HIV-1) or one (HMPV, MV) day later the percentage of GFP-expressing cells was determined by flow cytometry. In all experiments Pam3CSK4, Pam-Cys-SK4 and PHCSK4 treatment resulted in significantly higher infection percentages (p<0.05 using posthoc Dunnett's correction for multiple comparisons). Error bars represent the standard deviation of triplicates. A representative experiment out of three is shown.

Previously, it has been described that Pam3CSK4 enhances infection with or transmission of HIV-1 [28]–[30]. Here we show that the non-TLR agonists Pam-Cys-SK4 and PHCSK4 are equally capable of enhancing HIV-1 infection of a Jurkat-CCR5 cells as Pam3CSK4 (p<0.05, Figure 5A), indicating that also for HIV-1 TLR signaling-independent enhancement of infection can be observed. The same could be shown for two viruses from the same family as RSV: HMPV and two different MV strains (p<0.05, Figure 5B–D). For MV we also performed a virus binding assay and were able to show that, similar as described above for RSV, the enhancement was indeed mediated by enhancement of MV binding to their target cells (Figure S2). These viruses share membrane fusion as a common entry mechanism [39], but contain different attachment proteins and target different cellular receptors, suggesting that enhancement of virus binding is based on interactions between lipopeptides and biomembranes or common membrane-associated structures rather than with specific cellular or viral proteins.

### Interactions between cationic lipopeptides and GAGs explain inhibition of RSV infection in A549 and HEp-2 cells

**Figure 6:**
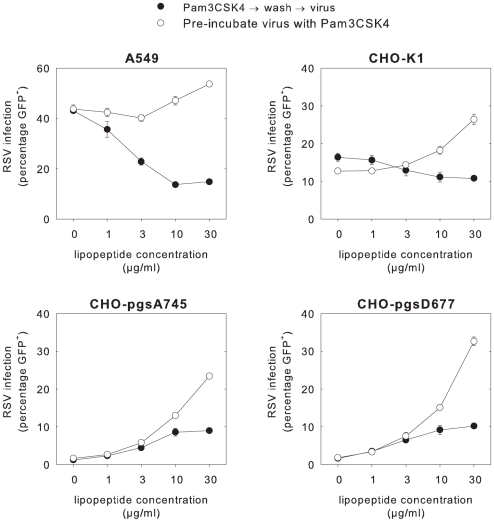
Mechanism of reduced rgRSV infection of A549 cells in relation to GAG expression. A549, CHO K-1, CHO pgsA-745 or CHO pgsD-677 cells were infected with rgRSV after pre-incubation of the cells (black symbols) or the virus (white symbols) with Pam3CSK4 (10 µg/ml) for 30 minutes at 37°C. In the GAG-expressing cell lines A549 and CHO-K1 pre-incubation of cells with Pam3CSK4 resulted in reduction of RSV infection, but in the GAG-deficient cell lines CHO-pgsA745 and CHO-pgsD677 this same condition resulted in enhancement of infection (p<0.01 in multiple Student's t-test with Bonferroni correction). Data are presented as percentages GFP-positive cells (means ± SD of triplicates). A representative experiment out of three is shown.

As described above and shown in Figure 1, and in sharp contrast to the reproducible enhancement of RSV infection in undifferentiated NHBE cells, BAL cells, dendritic cells and B-LCL, Pam3CSK4 inhibited RSV infection in the epithelial cell lines A549 and HEp-2. An important difference between these cell lines and the other cell types used in this study is the high expression level of GAGs in the immortalized epithelial cell lines A549 and HEp-2. GAGs are negatively charged long unbranched polysaccharides associated with proteoglycans (chondroitin-, dermatan-, heparan- and keratansulfate) expressed on cell membranes. It has been described that rgRSV and other laboratory-adapted RSV strains can use GAGs as cellular receptor, although this is considered an *in vitro* artifact and these molecules are not used as cellular receptors *in vivo* [40]. To address the role of GAGs in the observed Pam3CSK4-mediated inhibition of rgRSV infection in epithelial cell lines, we used Chinese hamster ovary cell (CHO) mutants deficient in GAG synthesis [40]. Wild-type and two GAG-deficient CHO cell lines (CHO-pgsA745 completely deficient in GAGs and CHO-pgsD677 deficient for heparan sulfate) were tested at the same multiplicity of infection (MOI) in two conditions. As expected, the percentage of rgRSV infection in A549 and wild-type CHO in the absence of lipopeptide was significantly higher than in the mutant CHO cells (p<0.01). Pre-incubation of cells with Pam3CSK4 decreased RSV infection levels in A549 and wild-type CHO cells, but enhanced infection in the GAG-deficient CHO cells (Figure 6). If the virus was pre-treated with the lipopeptide instead of the cells, enhancement was observed in all four cell lines (Figure 6). These findings demonstrate that Pam3CSK4-mediated inhibition of rgRSV infection in immortalized epithelial cell lines is related to interactions between the cationic lipopeptides and the negatively charged GAGs.

## Discussion

In the present study we have shown that Pam3CSK4 and at least two other structurally-related cationic lipopeptides can enhance infections with RSV, HIV-1, HMPV or MV independent of TLR signaling. By using rgRSV infection of B-LCL as a model system, we have shown that these lipopeptides can bind directly to both cells and virus, and strongly enhance virus binding to target cells. In contrast, Pam3CSK4 reduced rgRSV infection levels of A549 and HEp-2 cells under specific conditions, due to interactions with the negatively charged GAGs expressed on the surface of these cells.

Pam3CSK4 is a synthetic tripalmitoylated bacterial lipopeptide and is a potent activator of the NF-kB pathway, by forming an m-shaped heterodimer of TLR1 and TLR2 [41]. It has been described that Pam3CSK4 enhances infection or transmission of HIV-1 in different systems [28]–[30]. Our data show that Pam3CSK4-mediated enhancement of infection is not specific for HIV-1, but can also be shown for a number of other enveloped viruses including RSV, HMPV and MV. By blocking either binding to TLR1/2 or TLR1/2 signaling, and by using enhancing lipopeptides that do not activate TLR1/2, we demonstrate that this enhancement of RSV infection is independent of TLR signaling. Although we cannot exclude a role for TLR stimulation in HIV-1 transmission by Langerhans cells, our observations in Jurkat cells that Pam-Cys-SK4 and PHCSK4 showed a similar enhancement of HIV-1 infection as Pam3CSK4 demonstrate that also for HIV-1 TLR-signaling-independent enhancement of infection can be observed, and is likely related to a similar mechanism as shown for RSV and MV.

Using different lipopeptides we identified the cationic properties and the N-palmitoylated cysteine in Pam3CSK4 as crucial characteristics for enhancement of virus infection. We suggest that the N-palmitoylated cysteine may function as a structure interacting directly with the viral or target cell membrane. It has previously been described in the murine B-cell leukemia line BCL1 that already 20 minutes after incubation with Pam3-Cys-Ser a major amount of the lipopeptide can be found in the plasma membrane [42]. After membrane binding by Pam3CSK4 the exposed cationic SK4 sequence could subsequently interact with negatively charged structures on the membrane of virus or cell, and result in infection enhancement similar to what has been described for other cationic agents such as DEAE-dextran, polybrene or semen-derived enhancer of viral infection (SEVI) [37], [38], [43]. Interestingly, we found that highly sulfated polyanions could abrogate Pam3CSK4-mediated enhancement of rgRSV infection or binding, in a pattern almost completely identical to that described for the interaction between these compounds and SEVI [38]. However, additional non-electrostatic forces may be of importance, as two other lipopeptides containing positively charged amino acids, Pam3CSH4 and Pam3CSR4, could not mediate enhancement of RSV infection.

Pam3CSK4 was found to decrease RSV infection in epithelial cell lines A549 and HEp-2. However, this effect was only observed when the cells were pre-incubated with the lipopeptide, and not when the virus was pre-incubated with the lipopeptide. It has been described that rgRSV can efficiently bind to GAGs expressed on immortalized epithelial cell lines [44]. This effect is generally considered an *in vitro* artifact, as wild-type RSV strains cannot use GAGs as cellular receptors [40]. We reasoned that pre-incubation of A549 cells with the cationic Pam3CSK4 may reduce virus binding to the negatively charged GAGs, thus interfering with the normal attachment of RSV to these cells. Using a GAG-independent virus (MV) we could not detect reduced infection levels in A549 cells pre-incubated with Pam3CSK4 (data not shown). In order to test our hypothesis we performed RSV infections in normal or GAG-deficient CHO cells and indeed measured inhibition on pre-incubated CHO-cells expressing GAGs, but not in GAG-deficient CHO-cells. Since Pam3CSK4 not only enhanced infection of rgRSV but also of wild-type RSV isolates in B-LCL and primary epithelial cells, and this lipopeptide also enhanced infections with HIV-1, HMPV, and MV, we believe that the inhibition of rgRSV infection in A549 and Hep-2 cells is solely related to the laboratory adaptation of rgRSV to efficiently bind GAGs, and does not reflect potential interactions between bacterial lipopeptides and wild-type RSV infection *in vivo*.

Other cationic compounds, such as DEAE-dextran, polybrene, RANTES, and SEVI, have also been shown to enhance viral infection [38], [43], [45]. Polybrene, a synthetic cationic polymer neutralizing the charge repulsion between the virus and the cell surface, leads to enhancement of infection of a variety of retroviruses, including HIV [46]. SEVI enhances HIV infection by cross-linking HIV viruses to target cells, facilitating subsequent fusion [38], [45]. RANTES, a cationic chemokine, has been reported to enhance HIV-1 infection by binding to proteoglycans on the surface of target cells [47]. Interestingly, RANTES has also been associated with inhibition of RSV infection in HEp2-cells [48], which could be mediated through an analogous mechanism to Pam3CSK4-mediated reduction of rgRSV infection in A549 cells described here.

Pam3CSK4 is a synthetic mimic of the N-terminal parts of lipopeptides present in the cell walls of gram-positive and gram-negative bacteria, which have been identified as TLR agonists [49]. Therefore, the biological property of these lipopeptides as potential enhancers of RSV binding could explain in part the observed interactions between RSV and pneumococci [18], [19]. We speculate that carriage of respiratory bacteria in the upper or lower respiratory tract could facilitate binding of RSV to their target cells and thus play a role in the pathogenesis of severe RSV bronchiolitis. This would be a possible explanation for the fact that polyvalent pneumococcal vaccination has been associated with reduced RSV hospitalization rates [13]. Our results indicate that APCs are normally relatively resistant to RSV infection. However, lipopeptides such as Pam3CSK4 strongly enhanced rgRSV infection of these cells. Increased binding of RSV to and infection of APCs may boost immune activation and might as such be involved in pulmonary disease during RSV and bacterial co-infections.

Pam3CSK4 has been evaluated in several studies as a potential adjuvant for peptide vaccines [50]–[52]. Interestingly, our data suggest that the lipopeptide could also be considered as a potential adjuvant for live-attenuated virus vaccines. Development of a live-attenuated RSV vaccine has been hampered by difficulties in finding a proper balance between attenuation and immunogenicity of candidate vaccine viruses [53]. Pam3CSK4 has the potential to enhance binding of a live-attenuated RSV vaccine administered intra-nasally, while also stimulating innate immune responses through TLR1/2 interactions. However, as RSV vaccines will need to be given to very young infants, this latter property may be undesired. Alternatively, the non-TLR agonists Pam-Cys-SK4 or PHCSK4 could be considered as adjuvants, resulting in enhanced binding of an over-attenuated live-attenuated RSV vaccine without resulting in immune stimulation. Currently, we are performing studies in animal models to test this hypothesis.

In conclusion, we have shown that Pam3CSK4 and at least two structurally related cationic lipopeptides can enhance infections with RSV, HIV-1, human metapneumovirus and measles virus. In contrast to our initial hypothesis, this effect proved to be independent of TLR1/2 signaling but was mediated by enhancing binding of the viruses to their target cells.

## Materials and Methods

### Ethics statement

Human BAL cells were collected from patients suspected for pulmonary sarcoidosis in the framework of another study [54]. After written informed consent patients underwent fibre-optic bronchoscopy. The protocol was approved by the Medical Ethical Committee of the Erasmus University, Rotterdam.

### Cells

A549 cells (human pneumocyte type II carcinoma cells, ATCC CCL-185) and Epstein-Barr virus-transformed human B-LCL [55] GR were cultured in RPMI-1640 medium (Lonza) supplemented with L-glutamine, penicillin, streptomycin (Lonza) and 10% fetal bovine serum (FBS, Sigma-Aldrich). HEp-2 cells (human nasopharyngeal carcinoma cells, ATCC CCL-23) were cultured in Dulbecco's Modified Eagle Medium (Lonza) supplemented with L-glutamine, penicillin, streptomycin and 10% FBS. Monocyte-derived immature DCs were cultured as described [56]. In short, human peripheral blood monocytes were isolated from buffy coats (provided by Sanquin Blood Bank South West Region, Rotterdam) by density centrifugation, followed by selection of CD14^+^ cells using magnetic beads (MACS, Miltenyi Biotec GmbH). Purified monocytes were cultured in RPMI-1640 medium supplemented with L-glutamine, penicillin, streptomycin and 10% FBS and differentiated into immature DCs in the presence of IL-4 and GM-CSF (500 and 800 U/ml, respectively; Schering-Plough). Human broncho-alveolar (BAL) cells were cultured in RPMI-1640 supplemented with antibiotics and 10% FBS. NHBE cells (Clonetics) were used undifferentiated. Cells were cultured in 30 µg/ml type I collagen- (Nutacon) and 10 µg/ml fibronectin-, (Sigma-Aldrich) coated (culture flasks or flat-bottom 96-well plates (Corning) in bronchial epithelial cell basal medium (Clonetics) supplemented with penicillin and streptomycin. CHO K-1 cells (ATCC CCL-61), CHO pgsA-745 cells (ATCC CRL-2242) and CHO pgsD-677 cells (ATCC CRL-2244) were cultured in Ham's F12K medium supplemented with 10% FBS, penicillin and streptomycin. Jurkat T cells expressing CCR5 were previously described [28] and cultured in RPMI-1640 medium (Lonza) supplemented with 4500 mg/L glucose, 110 mg/l sodium pyruvate, 4 mM L-glutamine, 10% FBS, penicillin and streptomycin.

### Viruses

To generate HIV-1-GFP, 293T cells were transfected with the NL4.3-eGFP-BaL proviral plasmid (4 µg; generously provided by C. Aiken, Vanderbilt University, Tennessee, USA). After 3 days the NL4.3-eGFP-BaL virus was harvested and titrated using the indicator cells TZM-blue (contributed by John C. Kappes, Xiaoyun Wu [both at University of Alabama, Birmingham, Alabama, USA], and Tranzyme Inc. through the NIH AIDS Research and Reference Reagent Program). HMPV-EGFP (strain 00-1) was grown as described previously [57] and had a titer of 6.3×10^6^ TCID_50_/ml. Recombinant pathogenic and attenuated MV expressing EGFP were grown as described previously [58], [59]. The titers of recombinant pathogenic and attenuated MV were 1×10^6^ TCID_50_/ml and 6.3×10^4^ TCID_50_/ml, respectively. rgRSV is a recombinant virus based on RSV strain A2, which was a kind gift of Dr. M.E. Peeples and Dr. P.L. Collins. The construction and rescue of rgRSV has been described in detail elsewhere [32]. Briefly, GFP was inserted as the first, promoter-proximal gene in a full-length cDNA of the RSV A2 antigenomic RNA. As a consequence rgRSV-infected cells express GFP upon infection. rgRSV has been described to have the same growth kinetics and virological characteristics as the RSV A2 strain [32], [40]. rgRSV was grown and titrated on HEp-2 cells and aliquots were stored at −80°C until use. The titer of the virus used in these studies was 1×10^7^ TCID_50_/ml. Other (non recombinant) RSV strains used were the RSV subgroup A strains RSV Long (ATCC VR-26) and RSV A2 (ATCC VR1302), the RSV subgroup B strain 9320 (ATCC VR955) and three wild-type RSV isolates from our hospital archives, which were used at passage 2 in HEp-2 cells.

### Lipopeptides

Pam-Cys-SK4, PamDhcSK4, PamSK4, Pam2CSK4, Pam3CSE4, Pam3CSH4, Pam3CSK2, Pam3CSK4, Pam3CSK5, Pam3CSP4, Pam3CSR4, PHCSK4, Pam3CSK4-CF and CSK4-CF were synthesized by EMC microcollections, Germany (see Figure S1 for molecular structures). Stocks were prepared in distilled water at 1 mg/ml.

### TLR agonist experiment

A549, HEp-2, NHBE cells (2×10^4^ cells per well), monocyte-derived immature DCs, BAL cells or B-LCL (5×10^4^ cells per well in 96-well flat-bottom plates) were incubated with different TLR agonists (InvivoGen) for 6 hours as described previously [28]. Pam3CSK4, Pam2CSK4, purified LTA-SA, peptidoglycan from *S. aureus* (PGN-SA), lipopolysaccharide from *E. coli* (LPS) were incubated at final concentrations of 10 µg/ml and a synthetic di-acylated lipoprotein (FSL-1) and flagillin from *B. subtilis* at final concentrations of 1 µg/ml. Heat killed *L. monocytogenes* (HKLM) was used at a concentration of 1×10^8^/ml. After the 6 hour incubation, all cells were infected with rgRSV (1×10^4^ TCID_50_ per well for A549, HEp-2 and NHBE cells, 5×10^4^ TCID_50_ per well for immature DCs and 1×10^5^ TCID_50_ per well for BAL cells and B-LCL). 24 h post infection the cells were trypsinized (only for adherent cells) and percentages of GFP-positive cells were determined by flow cytometry (FACS Canto II, Becton-Dickinson).

### Experiments with lipopeptides only

5×10^4^ B-LCL were seeded in 96-well flat-bottom plates (V-bottom plates, if the cells were washed during the experiment). Unless stated otherwise, cells were pre-incubated with lipopeptides for 30 min before rgRSV was added (1×10^5^ TCID_50_), hereafter referred to as pre-incubation on cells. In some experiments, virus was pre-incubated with Pam3CSK4 for 30 min before addition to the cells, hereafter referred to as pre-incubation of virus. In these experiments, the virus was pre-incubated in a small volume and subsequently diluted at least 1∶10 before addition to the cells. 20–24 h post infection the percentages of GFP-positive cells were determined by flow cytometry (FACS Canto II, Becton-Dickinson).

### MyD88 experiment/TLR1/2 blocking

5×10^4^ B-LCL cells were plated in 96-well v-bottom plates. The cells were either pre-incubated with the myeloid differentiating factor 88 (MyD88) homodimerization inhibitory peptide (100 µM, Imgenex, USA) for 24 h or with polyclonal blocking antibodies to TLR1, TLR2, TLR4 or the combination of TLR1 and TLR2 (20 µg/ml, InvivoGen, USA) for 30 minutes. The cells were then incubated with Pam3CSK4 and subsequently infected with rgRSV (1×10^5^ TCID_50_). 20–24 h post infection the percentages of GFP-positive cells were determined by flow cytometry.

### RSV binding assays

To assess lipopeptide-mediated enhancement of RSV binding to cells, B-LCL (3×10^4^) were incubated with rgRSV (2.2×10^5^ TCID_50_) that had been pre-incubated (30 minutes, 37°) with lipopeptide (10 µg/ml). After 15 minutes at 37°C, cells were washed and incubated with FITC-labeled anti-RSV or anti-influenza-B as a control (Imagen immunofluorescence tests, OXOID, diluted 1∶10 in complete RPMI).

To assess binding of lipopeptides to RSV, lipopeptides were coated on high-binding 96-wells flat-bottom ELISA plates at a concentration of 300 ng/well in acetate buffer (pH 4.6) for two hours (37°C), after which free binding sites were blocked for one hour (37°C) with DMEM supplemented with 10% FBS. After washing, wells were incubated with RSV-A2 or with measles virus strain Edmonston as a control (both 10^6^ TCID_50_ per well) as control (1 hr, 37°C), followed by washing and addition of anti-RSV antibody (NIH clone 1107). ELISA plates were subsequently stained with goat-anti-mouse peroxidase and TMB as a substrate, and extinctions were determined at 450 nm.

To assess binding of lipopeptides to cells, B-LCL were incubated with different concentrations of carboxyfluoresceine-labeled Pam3CSK4 (Pam3CSK4-CF, Mw 2094) or CSK4 (CSK4-CF, Mw 1079) for 30 minutes at 4°C or 37°C. Subsequently, cells were washed twice and fluorescence was measured by flow cytometry. Results are shown as the geometric mean fluorescence (mean ± SD of triplicates).

### Polyanion experiments

Polyanion reagents (heparin, dextran sulfate, and chondroitin sulfate) were obtained from Sigma-Aldrich. Oversulfated heparin, de-O-sulfated heparin and oversulfated chondroitin sulfate were obtained from Neoparin. The polyanions were stored in PBS at a concentration of 30 mg/ml at −20°C. 5×10^4^ B-LCL were plated in 96-well v-bottom plates. The cells were pre-incubated with different lipopeptides for 15 minutes at 37°C and subsequently incubated for 15 minutes with the indicated polyanions (30 µg/ml). The cells were washed three times and infected with rgRSV (1×10^5^ TCID_50_).

### Enhancement studies with HIV-1, HMPV and MV

5×10^4^ Jurkat-CCR5 cells were plated in 96-well u-bottom plates and pre-incubated with different lipopeptides and subsequently inoculated with NL4.3-eGFP-BaL at different concentrations in the presence or absence of the indicated lipopeptides (all at 10 µg/ml). Two days post infection the cells were fixed and percentages of EGFP-positive cells were determined by flow cytometry. The other viruses were tested in B-LCL, using a single MOI and a concentration range of lipopeptide as described above, and determining percentages GFP-positive cells 20–24 hours post infection.

### Statistical analysis

All experiments were performed at least three times using triplicate measurements, except for the ELISA experiment with lipopeptides, which had duplicate measurements. Data are expressed as means ± standard deviations. Differences between two groups (treatment and control) were analyzed with Student's t-test, using Bonferroni's correction to correct for multiple comparisons. When comparing multiple treatment groups to the same control, differences were compared using 1-Way ANOVA with Dunnett's Correction for multiple comparisons. A two-sided (uncorrected) p-value<0.05 was considered statistically significant. Methods for specific statistical comparisons are given in the figure legends.

## Supporting Information

Figure S1 Molecular structures of (lipo)peptides used in this study.
(0.32 MB TIF)

Figure S2 Binding of measles virus (MV) pre-incubated with different lipopeptides to Vero cells
(1.19 MB TIF)
