# Supplementary figures and images for: The Synthetic Bacterial Lipopeptide Pam3CSK4 Modulates Respiratory Syncytial Virus Infection Independent of TLR Activation
Source: PLoS Pathog. 2010 Aug 19;6(8):e1001049. doi: 10.1371/journal.ppat.1001049 (PMC2924323; doi:10.1371/journal.ppat.1001049)

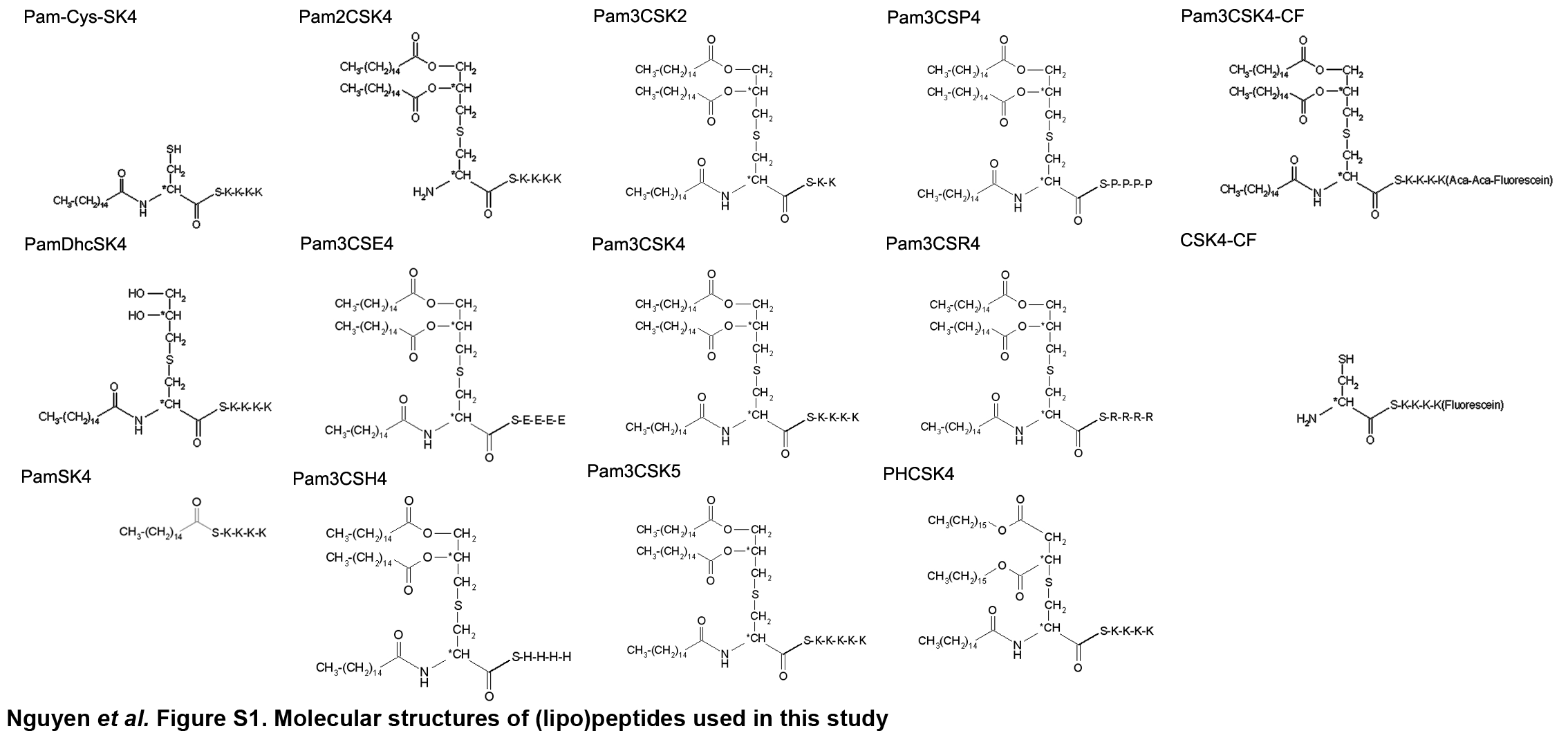

Supplement: Figure S1 — Molecular structures of (lipo)peptides used in this study. (0.32 MB TIF) [file ppat.1001049.s001.tif]

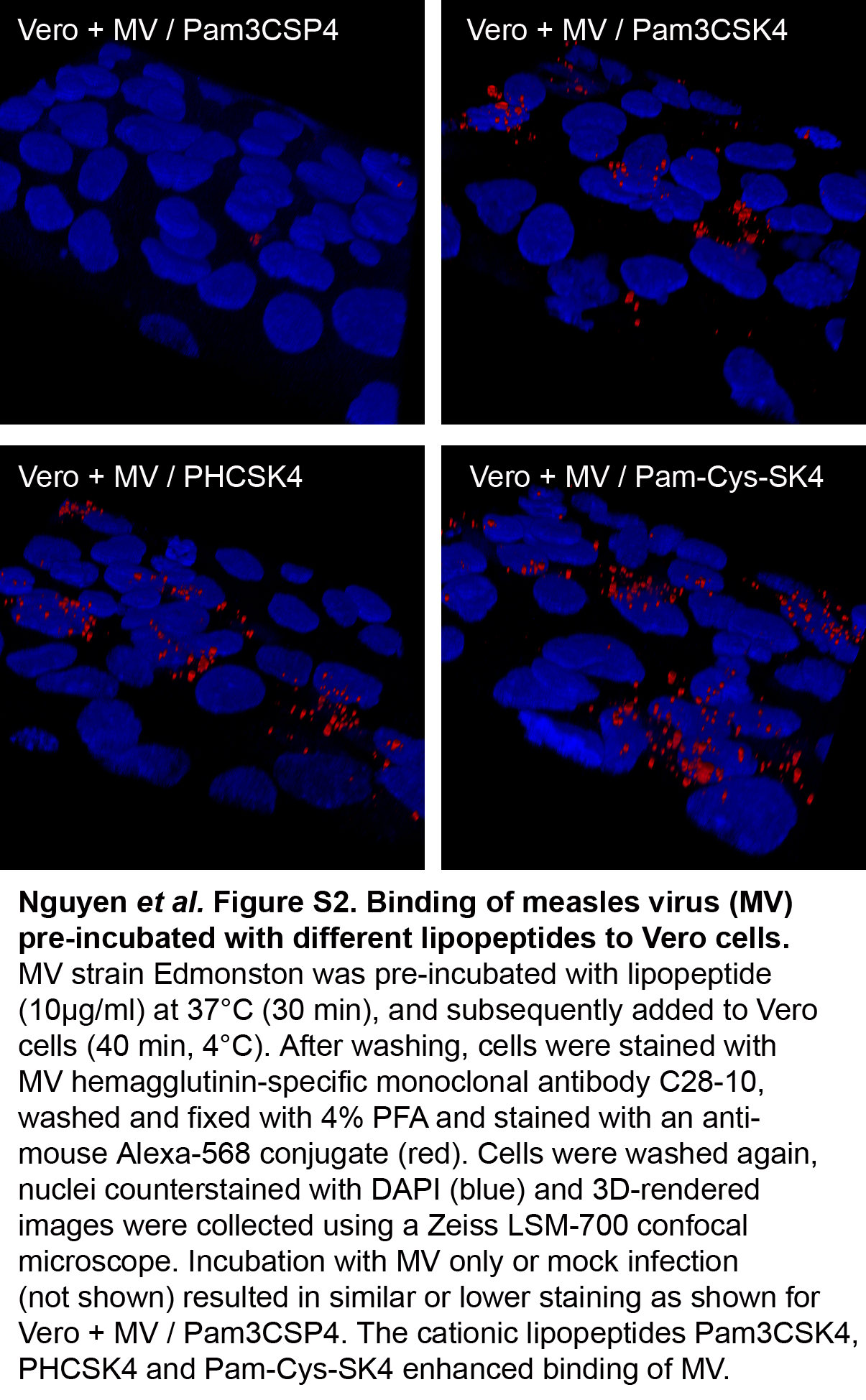

Supplement: Figure S2 — Binding of measles virus (MV) pre-incubated with different lipopeptides to Vero cells (1.19 MB TIF) [file ppat.1001049.s002.tif]
